# The immunological landscape of antibody-drug conjugates: from target to payload biology

**DOI:** 10.3389/fimmu.2026.1964101

**Published:** 2026-10-27

**Authors:** Jeremy D. Waight, Maria C. Speranza, Christopher B. Hopson, Kenneth W. Hance

**Affiliations:** 1 GSK Oncology R&D, Collegeville, PA, United States; 2 GSK Oncology R&D, Waltham, MA, United States

**Keywords:** ADC-immune checkpoint inhibitor (ICI) combinations, antibody drug conjugate (ADC), Fc engineering, immunogenic cell death (ICD), tumor microenvironment (TME)

## Abstract

Antibody-drug conjugates (ADCs) have the potential to impact biology well beyond targeted cytotoxin delivery. Here, we discuss considerations for ADC immune interplay, from Fc biology to elicitation of immunogenic cell death (ICD), to manipulation of the tumor microenvironment. In recent years, antibody-drug conjugates (ADCs) have garnered significant attention as a major therapeutic modality in oncology. By combining a targeting mechanism with various classes of small-molecule payloads, ADCs offer a highly modular approach to either selectively deliver (or promote accumulation of) chemotherapeutic agents to tumors, fueling a dramatic expansion in ADC clinical development. With hundreds of candidates now progressing through clinical testing in solid tumors and hematologic malignancies, a more nuanced understanding of ADC biology is starting to take shape. Despite the relatively simplistic design, even modest changes to the antibody and/or warhead-linker-payload (WLP) design can result in significant biological variance. Instead of just scaffolds for drug delivery, a growing body of evidence supports a multifaceted role for ADCs, including a mechanistic relationship with the immune system. Here, we discuss considerations for ADC immune interplay, from Fc biology to elicitation of immunogenic cell death (ICD), to influence on the tumor microenvironment (TME). Finally, we explore the translational challenges of optimizing ADC–immunotherapy combinations, including payload selection, linker design, and safety considerations for combination regimens. Collectively, this review explores the evolving understanding of immunomodulatory characteristics of ADCs and their potential to bridge targeted therapy and cancer immunology.

## Graphical Abstract

**Figure:**
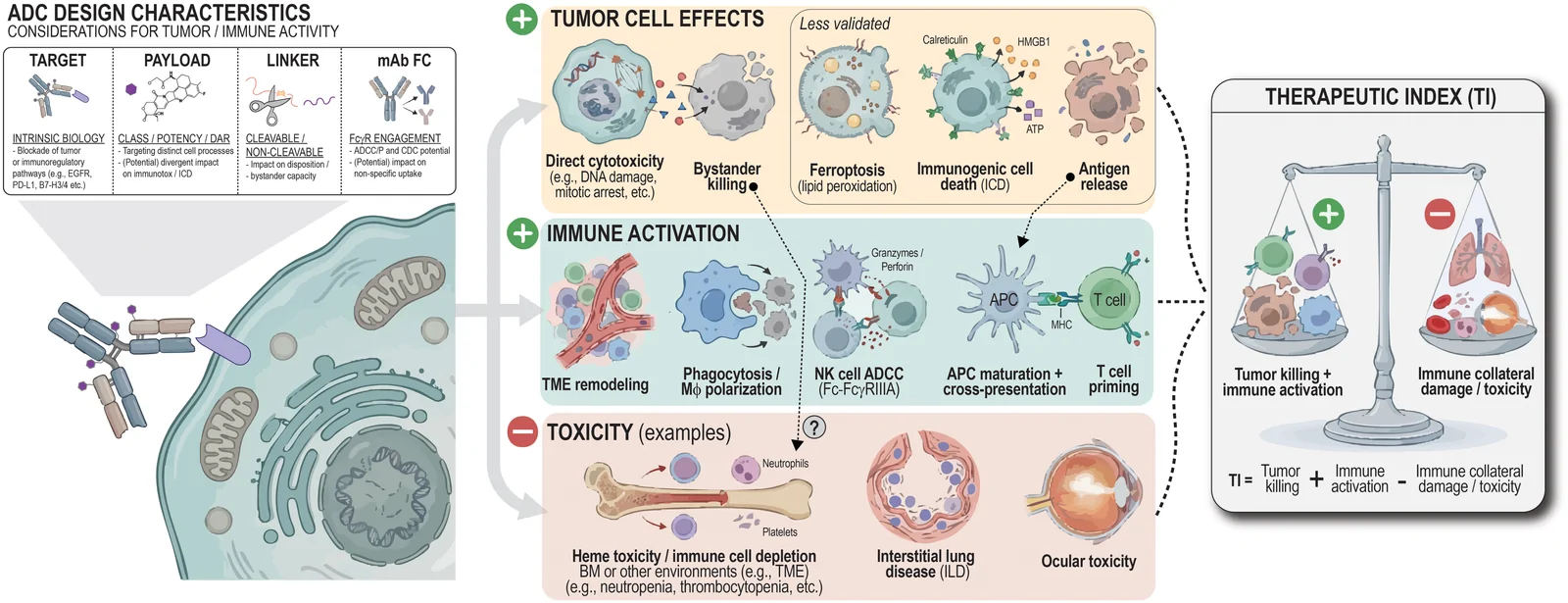
Overview of the impact of ADC design characteristics on tumor cells, potential immune interplay, and the balance of positive versus negative functional effects in cancer patients (i.e., the therapeutic index). Example design characteristics include target selection, inter- and intra-class payload biology (and DAR), linker, and antibody Fc selection. Each of these characteristics may differentially influence antitumor activity and/or toxicity, whether cytotoxic or immune-related. DAR, drug-antibody ratio; mAb, monoclonal antibody; HMGB1, High Mobility Group Box 1; ATP, adenosine triphosphate; Mϕ, macrophage; APC, antigen presenting cell; ADCC, antibody-dependent cellular cytotoxicity.

## Introduction

As a prominent and evolving therapeutic modality, ADCs represent a paradox of simplicity and complexity that continues to provide meaningful benefit to cancer patients. Over the past 5 years the number of regulatory approvals for ADCs and landmark clinical trials have expanded rapidly, with several prominent examples across hematological malignancies (e.g., loncastuximab tesirine in refractory large B-cell lymphoma), solid tumors (e.g., mirvetuximab soravtansine in platinum-resistant ovarian cancer [PROC] and trastuzumab deruxtecan in breast cancer) (1). This proliferation of non-clinical and clinical research has brought about waves of innovation that appear modest on the surface but have provided substantial impact on patient outcomes. For example, linker-payload iteration from the microtubule inhibitor (MTi)-based trastuzumab-DM1 (T-DM1) to the topoisomerase 1 inhibitor (TOPO1i)-based trastuzumab-DXd (T-DXd) resulted in a dramatic survival benefit of HER2+ breast cancer patients in the DESTINY-Breast03 trial (median[m]PFS of 7.8 months versus 29.0 months, respectively) (2, 3). This observation, in turn, led to an exponential rise in the use of ADCs with cleavable linkers and TOPO1i payloads (1).

Beyond intrinsic design characteristics, other areas of innovation have started to take shape, including alternative approaches to ADC clinical trial design (e.g., perioperative studies, maintenance dosing, etc.), evaluation of novel combinations, or both (e.g., KEYNOTE-B15/EV-304 trial, NCT04700124). Indeed, combinations with divergent therapeutic modalities like immune checkpoint inhibitors (ICI) have started to provide tangible clinical benefits. One of the first ICI + ADC combination studies to demonstrate clinically meaningful benefit over standard chemotherapy was the EV-302 trial, which evaluated the MMAE (MTi)-based NECTIN-4 ADC enfortumab vedotin (EV) with pembrolizumab (anti-PD-1 ICI) in treatment-naïve locally advanced or metastatic urothelial cancer (UC; NCT04223856) (4, 5). Compared to platinum-based chemotherapy, EV + pembrolizumab demonstrated substantial improvements in median progression-free survival and overall survival (12.5 vs. 6.3 months and 33.8 months vs. 15.9 months, respectively). A similar combination benefit was observed with the HER2-directed disitamab vedotin (DV, MMAE, MTi) and toripalimab (anti-PD-1 ICI) in HER2-expressing urothelial cancer (NCT05302284). Outside of UC, a growing number of clinical studies to evaluate ADC-ICI combinations are being conducted across hematological malignancies and solid tumors, of which we will catalog here. Clinical testing of ADC and immune-based combinations has also recently expanded beyond ICIs into targeted immunotherapy (T cell engagers, TCE) as well, with exemplars in both heme (NCT04970901, LOTIS-7) and solid tumors (NCT06898957, DeLLphi-310).

Despite this innovation, and the growing impact of ADCs in oncology, the predominant framework for understanding their mechanism of action remains centered on a canonical sequence of events: antigen binding, internalization, enzymatic processing, and cytotoxic effect. While mechanistically accurate, other ADC characteristics, including the impact of the antibody/target biology, immunological consequences of ADC-driven tumor cell death, and mechanistic interplay between different ADC payloads and the immune system remain poorly defined. While similar topics (e.g., ADC + immunotherapy combinations, ICD and TME remodeling) have been reviewed recently, here we provide a comprehensive overview of the potential immunological mechanisms of ADCs in cancer therapy (6–8). This exploration encompasses potential influence of Fc-FcγR co-engagement, payload-driven ICD induction, TME immunomodulation, clinical combinations (e.g., ICI and TCE), and read through to emerging novel payload platforms and/or dual payloads. We argue that, in many cases, the immunological properties of ADCs can influence their therapeutic activity and have implications for rational drug design, combination strategies, and therapeutic index.

## Impact of antibody and target biology: Fc-FcγR co-engagement with ADCs

ADCs are primarily comprised of a mono or multi-specific antibody, a warhead-linker, and a range of cytotoxic or immunostimulatory payloads. Just like warhead-linker and payload design, antibody selection comes with choices; not all of which are immunologically neutral. The main human IgG subclasses (IgG1, IgG2, IgG3, and IgG4) differ substantially in their capacity for engagement with activating (FcγRI, FcγRIIa, FcγRIIIa) and inhibitory (FcγRIIb) FcγRs, which impacts their ability to facilitate effector functions like antibody-dependent cellular cytotoxicity/phagocytosis (ADCC/P) and complement-dependent cytotoxicity (CDC) (9, 10). Human IgG1 and IgG4 antibodies are the most commonly utilized isotypes for ICI- and/or ADC-based applications, with IgG1 exhibiting the highest affinity for activating FcγRs and strongest ADCC/P/CDC activity (11). By contrast, IgG4 antibodies are considered as Fc-reduced, lacking substantial effector activity.

The vast majority of clinical-stage ADCs, including T-DXd, SG, and EV employ a standard IgG1 backbone. Preclinical evidence highlights the contribution of NK cell- and macrophage-mediated ADCC/P to T-DXd and T-DM1 (IgG1 backbone) antitumor activity (12–14). These findings should not come as a surprise, as the HER2 antibody trastuzumab, which serves as the backbone for many clinical-stage ADCs, has been well-characterized for its ability to facilitate ADCC/P and CDC (15, 16). In addition to natural isotypes, Fc-engineering has become more commonplace for attenuation or promotion of Fc-FcγR co-engagement or to enable certain conjugation and bi-specific approaches. Alternative isotypes and Fc-engineered variants (e.g., afucosylation [Fc-enhanced] or N297A/LALA-PG/aglycosylation [Fc-silent]) have started to make their way into late-stage (phase 2+) clinical testing (**Table 1**) (17).

**Table 1:** Snapshot of clinical-stage Fc-engineered ADCs.

Drug Name	Fc region	Target	Company
Belantamab mafodotin	Fc-enhanced	BCMA	GSK
ABBV-319	Fc-enhanced	CD19	AbbVie
Sofetabart mipitecan	Fc-silent	FOLR1	Eli Lilly
Vobramitamab tavatecan	Fc-silent	B7H3	Macrogenics
SYS6043	Fc-silent	B7H3	CSPC Pharma
SKB500	Fc-silent	B7H3	Kelun-Biotech
SKB518	Fc-silent*	PTK7	Kelun-Biotech
KIVU-107	Fc-silent	PTK7	Kivu Bioscience
KIVU-305	Fc-silent	CEACAM5	Kivu Bioscience
TUB-040	Fc-silent	NaPi2b	Tubulis
TUB-030	Fc-silent	5T4	Tubulis
IBI343	Fc-silent	CLDN18.2	Innovent Bio
HMBD-501	Fc-silent	HER3	Hummingbird Bio
FS001	Fc-silent	ITGA2	Foreseen Biotech
MGC028	Fc-silent	ADAM9	Macrogenics
FZ-AD005	Fc-silent	DLL3	Fudan-Zhangjiang Bio
SYS6005/RB-164	Fc-silent	ROR1	Radiance Biopharma

Snapshot of early-stage Fc-modified clinical-stage assets (cut off: September 1st, 2026). Other non-IgG1 ADCs, like gemtuzumab ozogamicin, Inotuzumab ozogamicin, BL-M11D1 (all IgG4), MT-8633 (IgG2), and SHR-1826 (IgG2) are also under active or prior clinical evaluation. *Unconfirmed. Inferred from Kelun-Biotech’s ADC platform.

Despite the increased presence of Fc-engineered ADCs in the clinical space, particularly Fc-reduced (e.g., Fc-silent) ADCs, little is known regarding the potential benefits or drawbacks of Fc-FcγR co-engagement. Like many other ADC characteristics, this adds to their overarching complexity driven by the level of versatility available for this modality. Fc-FcγR biology is best understood as a disposition characteristic for ADCs. That is, intact Fc can route ADCs into tumor associated macrophages/monocytes (TAM), peripheral myeloid sinks, megakaryocytes, or tissue resident macrophages. Whether this disposition helps or hurts depends on where the ADC is being processed and what payload is being released. Non-clinically, the clearest advantage is intratumoral macrophage-assisted efficacy (e.g., ADCC/P) and/or promotion of ICD related functional effects, whereas the clearest disadvantage is target-independent processing and payload release (e.g., alveolar macrophage-mediated lung inflammation) (18, 19). As should be expected, many of these observations are driven by the presence of effector populations, such as TAMs. For example, Li et al. demonstrated that a non-specific (hIgG1-directed) MMAE-based ADC elicited comparable tumor control to a tumor antigen-specific ADC in xenograft models; an effect that was contingent on the presence of TAMs (20). More recently, Tsao et al. showed that T-DXd, which is comprised of a wildtype hIgG1 antibody backbone, engages with FcγRs, leading to ADCP-driven antigen presentation and T cell activation. Moreover, the authors show that ICD elicited by the TOPO1i payload (DXd) promotes TLR4 and STING-dependent macrophage activation (14).

Although effector populations may contribute to ADC efficacy, only a small number of Fc-enhanced molecules have reached the clinic — namely belantamab mafodotin and ABBV-319 (**Table 1**). Both are designed for hematological malignancies and leverage ADCC activity as a payload-independent feature intended to enhance antitumor activity. However, while Fc-dependent antitumor activity has been shown non-clinically for these molecules, specific contribution of Fc effector function to clinical activity is still unknown (21, 22). Given the dearth of clinical-stage molecules, as well as the consolidation of these ADCs in hematological tumors — whose microenvironment differs markedly from that of solid tumors — a robust understanding of the potential benefits of Fc-enhancement remains a challenge. Generally, inferences have been, and continue to be, drawn by comparing Fc-enabled ADCs (e.g., WT IgG1) against the growing number of Fc-reduced/silent ADCs in clinical testing. Even so, the field is lacking sufficient pharmacodynamic (PD)-based studies to deconvolute contribution of components.

In contrast to the potential benefits that engagement of FcγR on effector populations may bring, alveolar macrophages and target-independent FcγR-mediated ADC uptake have been implicated in the promotion of pneumonitis/ILD (23). Recent non-clinical work by Koganemaru et al. demonstrated the impact of FcγR and alveolar macrophages on payload release in lung tissues (18). The authors found that while ADC payload release in tumor tissue correlated with antigen expression, payload release in lung tissue was driven by dose and was irrespective of target antigen expression. Using the THP-1 myeloid cell line (FcγRI+ and FcγRII+) as a surrogate for alveolar macrophages, they also found that Fc blockade significantly reduced non-specific payload release.

It should be noted that, when assessed by tissue immunostaining, lung macrophages have been shown to primarily express FcγRI and FcγRII (A/B not defined), rather than FcγRIIIA (24). This could suggest that common Fc-enhancement strategies that aim to increase FcγRIIIA binding and ADCC activity, whilst demonstrating minimal improvement in FcγRIIA binding, may not exacerbate non-specific uptake in the lung. All of this is premature, however, as Fc-enhanced ADCs with ILD-associated payloads (e.g., TOPO1i and eribulin) are lacking in the clinical setting (**Table 1**). To explore this hypothesis further, a potentially informative line of investigation could be the design and evaluation of Fc-enhanced ADCs in non-clinical safety models, including models of lung inflammation/ILD (23). Although purely speculative, the evaluation of TOPO1i-based ADCs harboring mutations to improve binding to activating FcγRIIA (G236A), FcγRIIIA (afucosylation or S298A/E333A/K334A) receptors or the inhibitory FcγRIIB (S267E/L328F) receptor may provide a clearer mechanistic framework for specific FcγR interactions and unintended toxicities (19, 25).

In addition to ILD, non-specific uptake of ADCs has been implicated in other adverse events, such as the liver or eye. Ocular toxicities have been documented for several clinical-stage ADCs, including belantamab mafodotin (belamaf, BCMA), tisotumab vedotin (TF), enfortumab vedotin (EV, NECTIN4), trastuzumab emtansine (T-DM1, HER2), and mirvetuxemab soravtansine (mirv, FOLR1) (26). Notably, all of these examples leverage different target antigens. While ADC-elicited adverse events are typically influenced by multiple factors, payload class appears to play a prominent role in the level of ocular toxicity observed in patients. Indeed, ADCs with significant ocular toxicities (e.g., belamaf, T-DM1, etc.) all harbor MTi-based payloads, whilst other non-MTi-based ADCs (e.g., T-DXd) demonstrate modest ocular AEs despite having a fully functional IgG1 Fc backbone (26). This is not a universal truth, as other payload classes (e.g., DNA-alkylating agents such as trastuzumab duocarmazine T-Duo]) have been tied to elevated ocular toxicities (27). However, while Fc selection may influence non-specific uptake of ADCs, the payload and sensitivity to that payload class remains a key determinant of toxicity (28).

In addition to the balance of monomeric Fc-FcγR interactions, aggregation of Fc-enabled ADCs has been proposed to promote antigen-independent cytotoxicity (29). Because linker-payload conjugation can have significant effects on ADC hydrophobicity and associated aggregation, one could speculate that linker-payload design would play a critical role in promoting or mitigating non-specific toxicity for Fc-enabled ADCs. Relevant to this, Aganati et al. recently showed that there were significant differences in the capacity of ADCs to elicit lung injury, even if they contain the same payload class (e.g., TOPO1i), an observation that has also been borne out clinically with DXd versus SN-38 (30). The authors found that linker-payload design was critical to inducing ILD-like effects. To test this, they generated a T-DXd tool ADC (T-MC-GGFG-DXd) alongside a similar “T-DXd-like” ADC carrying a non-cytotoxic cyclohexylamine dummy payload (T-MC-GGFG-Cy). Interestingly, both constructs elicited similar levels of lung injury, even though one lacked a cytotoxic payload — suggesting that overall ADC design, including the linker itself, can influence ILD risk independent of the payload’s cytotoxicity. These design characteristics are certainly something to monitor as the variety of clinical-stage ADCs expand. For example, correlations between alternative linker designs (e.g., rinatabart sesutecan’s branched hydrophilic linker-payload) or reduced permeability of TOPO1i payloads (e.g., belotecan) and ILD will be of significant interest going forward (31, 32).

While too early to make assertions, as datasets mature across assets, meta-analyses comparing efficacy and immunotoxicities for ADCs with divergent antibody variants will be critical for the field. This may be particularly interesting for assets with Fc differentiation, the same target, and exploration across multiple tumor types. As clinical data build, one example, B7H3, provides a potential opportunity to explore differences across Fc-enabled and Fc-silent molecules in many tumor types. Additionally, given the large number of TOPO1i-based ADCs utilizing identical targeting frameworks (e.g., HER2 ADCs: trastuzumab deruxtecan, -rezetecan, -bultecan, -pamirtecan, videtecan, etc.), large-scale analysis of clinical safety profiles may provide key insights into the impact of linker-payload design on off-target toxicities.

## ADCs and immunogenic cell death

Immunogenic cell death is a form of regulated cell death characterized by the release of danger-associated molecular patterns (DAMPs) that can activate innate immune cells (i.e., signal 0) and prime adaptive antitumor immunity (33, 34). However, DAMP release alone does not establish that ICD has occurred, and it is important to distinguish between DAMP exposure and immune stimulation. DAMP exposure, including cell surface calreticulin (CALR), high-mobility group box 1 (HMGB1), ATP, and type I interferon (IFN) release have primarily been described in the context of systemic chemotherapy, radiotherapy, photodynamic, and targeted therapies and is reflects a cell-intrinsic stress response that is frequently used as surrogate biomarker of ICD (35). On the other side, downstream evidence of immune stimulation, like APC maturation and antigen presentation, T cell priming and expansion and DC migration reflect the direct engagement on the immune system. With widespread clinical application and an underlying chemotherapeutic mechanism for many of these compounds, this area of investigation has expanded to ADCs. Given the multiple components of ADCs, including different payload classes and target antigen biology capable of eliciting ICD, a full understanding of the mechanistic interplay between ADC treatment, DAMP release, downstream immune activation and immune invigoration is complex. Descriptions of ADCs and elicitation of ICD markers and/or immune activation spans over a decade, with characterization of MTi-based ADCs, like brentuximab vedotin (CD30, MMAE), as some of the first examples (36). Using free payloads and MMAE-conjugated ADCs, Muller et al. showed that an intact host immune system was important for full therapeutic benefit of dolostatins/MTis. Through functional readouts rather than biomarker presence alone, they found that MMAE ADCs promoted antigen uptake and migration of DCs to tumor-draining lymph nodes. The authors extended these observations from lymphoma to HER2+ breast cancer using T-DM1, emphasizing the impact of ADC and ICI combinations on the number and activation of tumor infiltrating T cells as well as robust tumor rejection in tumors with limited sensitivity to the respective monotherapies (37). While it is now common practice to consider the interplay between a contemporary therapeutic modality (immunotherapy) and a burgeoning field (ADCs), broad application of ICI + ADC is still not fully adopted across the clinical landscape.

From an initial conceptual framework of the potential co-dependence between ADCs and the host immune system, a growing number of studies likely are being conducted on other classes of ADCs, including DNA damaging agents (7). With the rapid expansion in number and type of ADCs, and the initial descriptions of practice-changing drugs like T-DXd, functional characterization of ADC-induced ICD associated effects started to build across the field of ADC payloads. During this phase (2017-2019), PBD-, tubulysin-, and TOPO1i-based ADCs all demonstrated some level of immune-dependent efficacy, underscoring the potential ICD interplay as more than just a single payload class effect (38). Shortly after, ADCs with different payload classes were shown to induce DAMP release alongside evidence of APC activation, marking a methodological shift toward pairing DAMP/Biomarker readouts with functional immune correlates. It should be noted, however, that many “hallmark-driven” ADC-ICD claims in the field still rest primarily on marker expression rather than demonstration of a direct connection to immune stimulation (39). One nice example from Bauzon et al. not only underscored the differential enzymatic susceptibility of ADC linkers but also demonstrated the impact of a TOPO1i ADC on the induction of ICD markers (e.g., CALR, heat-shock [HSP] proteins, etc.) and APC activation (40). Tsao et al. took this a step further, by implicating Fc-FcγR co-engagement as functionally required for ICD-associated immune activation, rather than DAMP release alone, in the context of T-DXd (14).

Emergence of next-generation concepts such as dual-payload ADCs, degrader antibody conjugates (DACs), and novel single payload classes are critical to the progression of the field – and characterization of their capacity to induce ICD will go together. Because many of these next-generation payloads elicit cell stress and cytotoxicity, it’s predicted that they may also impart some level of ICD. However, the nuances in level of ICD, mechanistic pathway convergence, and combination potential will all be important to characterize. For example, N-myristoyltransferases 1 and 2 (NMT1/2) inhibitors have been implicated in single or dual payload ADCs and create significant multifaceted cellular stress and apoptosis, however, their overall impact (in an ADC format) on ICD hasn’t been looked at to the same level as MTi- or TOPO1i-based ADCs (41). An additional concept in the realm of next-generation ADCs is the use of dual payloads comprised of a mix of cytotoxin and immunomodulatory payloads (42). Depending on the nature of the immunostimulatory payload, this approach has the potential to not only promote adaptive immune responses but also impact innate effector populations prior to and during ICD events elicited by the cytotoxic payload (43).

Finally, an emerging area of research that is interrelated with ICD is the impact of ADC on tumor cell ferroptosis and lipid peroxidation (LPO) (44). Ferroptosis is a form of regulated cell death caused by iron-dependent LPO that is distinct from apoptosis and necrosis. Ferroptosis occurs when cellular antioxidant defenses, namely glutathione peroxidase 4 (GPX4), fail to control oxidative damage to membrane lipids, leading to loss of membrane integrity and cell death. Over the past few years, ferroptosis has become a focus in cancer, with growing interest in its interplay with various therapeutic modalities, including chemotherapy and ADCs (45). Kinetics may be an important factor for ferroptosis and ICD as oxidized lipids, which are known to suppress myeloid function and phagocytosis, accumulate during the late stages of ferroptosis (46). By contrast, early ferroptosis is associated with a strong vaccinal effect, releasing DAMPs and causing maturation of APCs (47, 48).

The concept of ADC-induced ferroptosis is built off prior descriptions with systemic chemotherapy (49). Many cytotoxic payloads, irrespective of their canonical target, can shift the balance in LPO by increasing reactive oxygen species (ROS), effectively lowering the ferroptosis threshold in target cells (50). While work specific to ADCs is limited, Koganemaru et al. demonstrated that the MTi-based FOLRα ADC MORAb-202 was able to elicit various hallmarks of ferroptosis in non-clinical models, including ACSL4 upregulation, GPX4 depletion, LPO, and cystine/cysteine metabolism (51). The authors linked this to ICD events, demonstrating HMGB1 release and subsequent recruitment of DCs and T cells. Ferroptosis/LPO has also been associated with non-canonical TOP cleavage complex (TOPcc) trapping for both topoisomerase I/II poisons, adding the repertoire of mechanisms by which TOPO-based ADCs can elicit replication stress (52).

In a recent study using non-clinical models, the antitumor activity of the TOPO1i-based B7H3 ADC DS-7300a (ifinatamab deruxtecan, I-DXd) was significantly improved when combined with an inhibitor of GPX4, underscoring the potential interplay between ferroptosis/LPO to ADC activity (53). Notably, this observation was in TP53-deficient tumors. Because p53 can lead to SLC7A11 repression, which decreases cystine uptake and sensitizes cells to ferroptosis, this provides a plausible mechanistic rationale by which TOPO1i signaling can lower the ferroptosis threshold, and supports the combination with GPX4/SLC7A11 inhibitors, particularly in a TP53-deficient setting (54).

Together, these findings suggest that ICD may serve as a mechanistic bridge between ADC-mediated cytotoxicity to durable antitumor responses. However, conclusions from these observations should be taken with caution, as isolating the contribution of ICD to durable clinical responses (e.g., PFS and OS) is inherently complex. Moreover, as stated previously, the presence of ICD markers on its own is not sufficient to establish functional consequence, and thus, dedicated clinical biomarker efforts like the recent belamaf analysis by Watson et al. are critical, and should be a priority focus for ADC development going forward (55).

## TME remodeling and immune toxicities by ADCs

In recent years, as the number of approved and clinically developed ADCs has expanded, the field has progressed beyond understanding ADC design, target engagement, and linker stability toward a broader understanding as to how these therapeutics interact with, and in some cases reshape, the TME (8).

A key limitation of targeted therapeutics, including ADCs, is that most clinically validated targets are tumor-associated rather than tumor-specific antigens. Because many of these antigens are also expressed on normal tissues with varying degrees, particularly hematopoietic and immune cell populations, ADCs carry a risk of on-target/off-tumor toxicity. This risk is especially relevant in hematological malignancies, where target antigens are often broadly shared between malignant cells and normal immune cells, frequently resulting in clinical myelosuppression (1, 28, 56). The hematopoietic system is particularly vulnerable for several reasons: (I) depletion of immune subsets can produce a range of clinical consequences, from increased susceptibility to infection to autoimmune dysregulation and, in severe cases, lethal immunosuppression; (II) target antigens are often broadly expressed across normal immune lineages; and (III) immune cells may be especially sensitive to cytotoxic payloads because of their high proliferation rate (28). It should be noted that, in heme malignancies, many of these common immune populations share the same primordial micro- and/or macro-environment with the tumor, such as the blood or the bone marrow.

This also extends to overlap with progenitor populations between healthy and malignant cells. For example, BCMA-directed belamaf (non-cleavable, MMAF; multiple myeloma), CD19-directed loncastuximab tesirine (PBD; large B cell lymphoma), CD22-directed inotuzumab ozogamicin (calicheamicin, B-ALL), and CD79b-directed polatuzumab vedotin (MMAE, B cell lymphoma) are all highly expressed in the indications for which they are approved (57–60). However, they are also present at various points of development for the B cell lineage, with some expressed on plasma cells (e.g., BCMA) or on B cell precursors (e.g., CD22), resulting in predictable on-target immune toxicities such as increased infection risk and hypogammaglobulinemia (56). Similar principles apply to myeloid-targeting ADCs, such as gemtuzumab ozogamicin (calicheamicin, AML) and pivekimab sunirine (IGN, blastic plasmacytoid dendritic cell neoplasm [BPDCN] and testing in AML), where CD33 and CD123 are expressed on multiple immune cells, causing on-target toxicities such as myelosuppression and cytopenias (61–64).

Further, ADCs targeting antigens expressed by T and NK cells also demonstrate the consequences of immune-lineage targeting. Brentuximab vedotin and camidanlumab tesirine, respectively CD30- and CD25-directed ADCs with MMAE and PBD payloads, have been developed to treat classical Hodgkin lymphoma (cHL) and other lymphomas. Because CD30 and CD25 are expressed not only on malignant cells but also on activated effector and regulatory T cells, treatment carries on-target risks beyond typical payload-related effects, such as Treg depletion, and increased risk of immune dysregulation or autoimmunity (65–67). Lorvotuzumab mertansine (DM1) is a CD56-targeted ADC that was developed for the treatment of several indications, including SCLC, MM, and Merkel cell carcinoma. While CD56 is highly expressed in tumors (e.g., >70% of SCLC), it is also a canonical marker for NK and NKT cells (68). In early-phase clinical study with etoposide/carboplatin, N = 10 infection-related deaths (pneumonia or sepsis) were reported and attributed to NK cell depletion, leading to immediate discontinuation of the program (69). Notably, although the lorvotuzumab mertansine program was discontinued, interest in CD56 as an ADC target has not disappeared, with a novel ADC (TOPO1i, DXC-006) recently entering clinical testing for SCLC and heme malignancies (70).

Beyond direct killing of antigen-expressing cells, bystander-capable ADCs can also influence the TME via indirect cytotoxicity (71). Many clinical ADCs harbor cleavable linkers and permeable payloads that allow cytotoxic agents to diffuse across cell membranes and kill neighboring cells irrespective of target antigen expression. While this property can offer a therapeutic advantage in highly heterogeneous tumors, it also introduces a potential liability: membrane-permeable payloads do not distinguish between antigen-negative tumor cells from antigen-negative immune effector cells within the TME. As a result, ADCs may cause collateral toxicity toward tumor-infiltrating immune cells (e.g., tumor-infiltrating T cells, NK cells, dendritic cells).

Within the TME, the most vulnerable immune populations are activated T and NK cells due to their proximity to antigen-positive tumor cells and intrinsic sensitivity of proliferating cells to chemotherapy (72). Indeed, T cell depletion/cytotoxicity induced by ADC can impact therapeutic efficacy, reinforcing the importance of immune interplay and underscoring the fine balance for ADC-based therapeutic index (39). This may be particularly relevant in immunologically rich, or ‘hot’, tumors, where exposure to excessive free payload may undermine the adaptive immune response and attenuate the combination potential between ADCs and immunotherapy. These findings demonstrate that the immunological impact of bystander killing is characterized by a delicate balance between tumor cell death and collateral depletion of effector immune cells; an effect that may be specific for each indication based immune composition within the tumor. Additionally, tumor-associated macrophages and/or myeloid cells (TAMs) can constitute up to 50% of the tumor mass, and can internalize, degrade and redistribute ADC-derived payloads (14, 73, 74). Thus, ADC-elicited depletion or ICD-based reprogramming of immunosuppressive myeloid cells/TAMs may have a significant impact on the overall immune landscape of the TME (20).

In contrast to the potential for incidental immunotoxicity, it could be expected that targeted destruction of tumor epithelium or stroma would allow for better infiltration of peripheral or parenchyma-associated immune populations into the tumor core (75). This physical ‘debulking’ effect may be particularly relevant for ADC targets that are restricted to tumor cells. However, for ADC targets that are also highly expressed on stromal populations (e.g., B7H3), the overall impact of specific ADCs on ‘chemical barriers’ to immune infiltration into tumors (e.g., immunorepulsion) may be an area of future mechanistic investigation (76).

Relative more traditional ADC formats, alternative designs, like antibody-nanoparticle conjugates (ACNPs), may offer additional opportunities to explore impact of targeted payload delivery to the TME (77). Like ADCs, ACNPs combine the specificity of antibody-mediated TME targeting with payload delivery. However, ACNPs offer the versatility of a nanoparticle core, allowing for a wider range of payload options (at exceptional DAR) and an enhanced permeability/retention (EPR) profile, providing a significant amount of optionality when it comes to the number and type of compounds delivered to the tumor. While still early in clinical development, it will be exciting to see what ACNPs offer in terms of both therapeutic benefit and TME remodeling.

## The evolving landscape of ADC and immunotherapy combinations

At a high level, combinations of multiple immune modulators targeting different immunological processes and/or enlisting distinct modalities (e.g., checkpoint inhibition versus induction of tumor cell death) have the potential to provide additional benefit to single agent/modality approaches (78).

In this review we discuss seminal studies, current trends, and future directions, however, several excellent reviews on ADC combinations and the evolving combination dogma for clinical assets have been published in recent years (7, 28, 79, 80). Available literature supports the combination of ADCs and ICI via multiple mechanisms including promotion of immunogenic cell death, the TME remodeling that cytotoxic payloads introduce, and context-dependent immune activation via FcγR engagement (81). Additionally, as noted previously, an intact immune system is not only necessary for PD-L1-driven antitumor responses but has also been shown to contribute to ADC activity (39). Support for combinations of ADC and immunotherapy agents goes beyond mechanistic rationale and non-clinical data, as combinations of ADCs and ICI have brought tangible benefits for cancer patients. Indeed, the number of late-stage clinical trials evaluating ADC and ICI combinations has been rapidly expanding in recent years, with a host of different MTi- and TOPO1i-based ADCs under investigation (**Table 2**).

**Table 2:** Snapshot of late-stage ADC and immunotherapy combinations (solid tumors).

ADC (Target/Payload)	ICI combo	Indication	Trial ID (Alias)	Notes/Learnings
EV (NECTIN4, MTi)	Pembro^a^	LA/mUC	NCT04223856 (KN-A39/EV-302)	• Standard setting ADC + ICI combo trial • Doubling of OS versus SOC (4, 5)^&^
EV (NECTIN4, MTi)	Pembro^a^	CI MIBC Perioperative	NCT03924895 (KN-905/EV-303)	• Perioperative EV-P + surgery in CI pts • 74.7% EFS vs 39.4% for surgery control (88)
EV (NECTIN4, MTi)	Pembro^a^	CE MIBC Perioperative	NCT04700124 (KN-B15/​EV-304)	• Perioperative EV-P in CE pts + cystectomy • 79.4% EFS vs 66.2% for cis–gem control (89) • Both regimens led to strong OS (>80%)
EV (NECTIN4, MTi)	Pembro^a^	MIBC	NCT07566156 (EV-309)	• EV/P vs cCRT in cystectomy-ineligible pts • Bladder- and RT-sparing option/implications • Final results not yet published
EV (NECTIN4, MTi)	Durva^b^; Treme	CI MIBC*	NCT04960709 (VOLGA)	• Perioperative EV-P + surgery in CI pts • Not only evaluates EV+ PD-L1, but also a triplet (EV + PD-L1 + CTLA-4) • Final results not yet published
EV (NECTIN4, MTi)	Pembro^a^	Adv. BC Time of Day	NCT07346053 (CHRONO-EVP)	• Time of day study (AM) vs (PM) EV-P • Implications for compliance, safety, efficacy • Final results not yet published
EV (NECTIN4, MTi)	Pembro^a^	LA/mUC + AGA	NCT07218380 (FORAGER-2)	• Tests a triplet (FGFR3i + EV-P) • Implications for targeted Tx (FGFR3) + EV-P • Final results not yet published
EV (NECTIN4, MTi)	Pembro^a^	LA/mUC	NCT06764095 (CAST-AI)	• Induction Tx (EV-P) + consolidation surgery • Evaluates exosomes and cfDNA for MRD • Final results not yet published
T-DXd (HER2, TOPO1i)	Rilve	BTC HER2+	NCT06467357 (DESTINY-BTC01)	• Combination of T-DXd + PD-1/TIGIT BiS • Implications for redefining 1L regimen • Final results not yet published
T-DXd (HER2, TOPO1i)	Pembro^a^; Rilve	EC MMRp HER2+	NCT06989112 (DESTINY-EC01)	• Tests both T-DXd + P and T-DXd + PD-1/TIGIT • Implications for redefining 1L regimen • Final results not yet published
T-DXd (HER2, TOPO1i)	Pembro^a^	NSCLC HER2+	NCT06899126 (DESTINY-Lung06)	• Tests T-DXd + P vs platinum + P in NSCLC • Intent to replace platinum in PD-L1 TPS<50% • Final results not yet published
T-DXd (HER2, TOPO1i)	Pembro^a^	GC/GEJC HER2+	NCT06731478 (DESTINY-Gastric05)	• Main cohort tests T-DXd + P in PD-L1 CPS ≥1 • Exploratory cohort of T-DXd in PD-L1 CPS <1 • Final results not yet published
T-DXd (HER2, TOPO1i)	Pembro^a^; Rilve	GC HER2+	NCT06764875 (ARTEMIDE-GC01)	• T-DXd + PD-1/TIGIT BiS • Intent to replace platinum + ICI/trastuzumab • Final results not yet published
SG (TROP2, TOPO1i)	Pembro^a^	LA/mTNBC	NCT05382286 (ASCENT-04)	• SG + P vs SOC chemo + P in PD-L1 CPS ≥10 • Led to FDA approval for SG + P in 1L • 11.2 mPFS vs 7.8mo for SOC control (90)
SG (TROP2, TOPO1i)	Pembro^a^	TNBC Adjuvant	NCT05633654 (ASCENT-05)	• Tests SG + P as an adjuvant Tx in pts with nodal/residual disease post-neoadj. Tx • Also assesses PD-(L)1 stratification • Final results not yet published
SG (TROP2, TOPO1i)	Pembro^a^	TNBC Neoadjuvant	NCT06081244 (ADAPT-TN-III)	• Neoadjuvant SG vs SG + P • Intent to replace platinum + IO/Trastuzumab • Final results not yet published
SG (TROP2, TOPO1i)	Pembro^a^	TNBC Neoadjuvant	NCT07178730 (ADAPT-TN-IV)	• Neoadjuvant SG + P vs chemo + P • Tests adding ADCs into KN-522 regimen • Final results not yet published
SG (TROP2, TOPO1i)	Pembro^a^	Adv. UC	NCT06524544	• Tests SG + P versus SOC chemo in patients who progress following PD-(L)1 and EV Tx • Setting novelty: evaluates an alternative payload class (TOPO1i) + P following lack of response to PD-(L)1 + EV (MTi) • Final results not yet published
SG (TROP2, TOPO1i)	Pembro^a^	mNSCLC^ No AGA	NCT05609968 (EVOKE-03)	• SG + P vs P in PD-L1 TPS ≥50% • Trial discontinued (non-significant mPFS improvement) • Final results not yet published
SG (TROP2, TOPO1i)	Zimberelimab^a^	NSCLC Adjuvant	NCT06431633 (ARIAN)	• Adjv SG + Z vs Z in patients without pCR • Also evaluates ctDNA correlations with OS • Final results not yet published
Sac-TMT (TROP2, TOPO1i)	Pembro^a^	mNSCLC^ No AGA	NCT06170788 (TroFuse-007)	• S-TMT + P vs P in PD-L1 TPS ≥ 50% • Unlike EVOKE-03, non-squamous only, and P dosed at Q6W vs Q3W • Final results not yet published
Sac-TMT (TROP2, TOPO1i)	Pembro^a^	LA/mBrC HR+/​HER2-	NCT06312176 (TroFuse-010)	• S-TMT + P vs S-TMT or SOC chemo • Potential novelty as a non-HER2 option in 1L • Final results not yet published
Sac-TMT (TROP2, TOPO1i)	Pembro^a^	TNBC	NCT06841354 (TroFuse-011)	• S-TMT + P vs S-TMT or SOC chemo • Tests ADC-ICI in in PD-L1 CPS <10 setting • Final results not yet published
Sac-TMT (TROP2, TOPO1i)	Pembro^a^	NSCLC Adjuvant	NCT06312137 (TroFuse-019)	• Adjv S-TMT + P vs P in patients without pCR following chemo-ICI + surgery • Final results not yet published
Sac-TMT (TROP2, TOPO1i)	Pembro^a^	mBrC/TNBC Neoadjuvant	NCT06966700 (TroFuse-032)	• Neoadjuvant S-TMT + P vs chemo + P followed by adjuvant chemo + P • Final results not yet published
Sac-TMT (TROP2, TOPO1i)	Pembro^a^	EC Maintenance MMRp	NCT06952504 (TroFuse-033)	• S-TMT + P vs P following chemo induction • Setting novelty: assesses ADC + ICI in pMMR, and as a maintenance treatment • Final results not yet published
Sac-TMT (TROP2, TOPO1i)	Pembro^a^	CC PD-L1+	NCT07216703 (TroFuse-036)	• Maintenance S-TMT + P + Bev vs P + Bev following SOC induction • Final results not yet published
Sac-TMT (TROP2, TOPO1i)	Pembro^a^	NSCLC PD-L1+ No AGA	NCT06448312 (OptiTROP-Lung05)	• S-TMT + P vs P in PD-L1+ tumors • Implications for 1L NSCLC SOC • mPFS benefit in PD-L1 TPS of 1-49% (NR vs 4.3 mo) and ≥50% (NR vs 9.5 mo) (91)
Sac-TMT (TROP2, TOPO1i)	Pembro^a^	NSCLC, PD-L1-, No AGA	NCT06711900 (OptiTROP-Lung06)	• S-TMT + P vs SOC chemo + P • PD-L1 TPS < 1% setting • Final results not yet published
Dato-DXd (TROP2, TOPO1i)	Durva; Pembro^a^	TNBC Adjuvant	NCT05629585 (TROPION-Breast03)	• Adjuvant D-DXd + Durva vs D-DXd or ICT in pts with residual invasive disease following neoadjuvant chemo + surgery • Final results not yet published
Dato-DXd (TROP2, TOPO1i)	Durva^b^	TNBC/BrC Neoadjuvant	NCT06112379 (TROPION-Breast04)	• Neoadjuvant D-DXd + Durva vs chemo + P and adjuvant Durva vs P (all ± chemo) • Final results not yet published
Dato-DXd (TROP2, TOPO1i)	Durva^b^	TNBC PD-L1+	NCT06103864 (TROPION-Breast05)	• D-DXd + Durva vs Dato-DXd or ICT + P • Tested in a PD-L1 CPS ≥ 10 setting • Final results not yet published
Dato-DXd (TROP2, TOPO1i)	Pembro^a^	NSCLC PD-L1+	NCT05555732 (TROPION-Lung07)	• D-DXd + P + platinum vs Dato-DXd + P or platinum + P in PD-L1 TPS < 50% • Intent to evaluate a platinum sparing option • Final results not yet published
Dato-DXd (TROP2, TOPO1i)	Pembro^a^	Adv. NSCLC PD-L1+ No AGA	NCT05215340 (TROPION-Lung08)	• D-DXd + P vs P in PD-L1 TPS ≥ 50% • Unlike EVOKE-03, non-squamous only • Final results not yet published
Dato-DXd (TROP2, TOPO1i)	Rilve	Adv. NSCLC PD-L1+ No AGA	NCT06357533 (TROPION-Lung10)	• D-DXd + Rilve vs Rive or P in PD-L1 TPS ≥50% • Similar design as TROPION-Lung08, but with PD-1/TIGIT BiS • Final results not yet published
Dato-DXd (TROP2, TOPO1i)	Rilve	NSCLC Adjuvant	NCT06564844 (TROPION-Lung12)	• Adjuvant D-DXd + Rilve vs Rive or SOC • Following complete surgical resection and ctDNA positivity • Final results not yet published
Dato-DXd (TROP2, TOPO1i)	Durva^b^	Adv. NSCLC No AGA	NCT05687266 (AVANZAR)	• D-DXd + Durva + platinum vs chemo + P • Final results not yet published
T-Rez (HER2, TOPO1i)	Adebrelimab^b^	TNBC PD-L1+	NCT07111832 (SHR-A1811-313)	• T-Rez + Adebreli vs Tor + chemo • Final results not yet published
T-Rez (HER2, TOPO1i)	Adebrelimab^b^	GC/GEJC	NCT07118527 (SHR-A1811-316)	• T-Rez + Adebreli + chemo vs Trastuzumab + chemo + P • Final results not yet published
T-Rez (HER2, TOPO1i)	Adebrelimab^b^	TNBC Adjuvant	NCT07679360 (SHR-A1811-318)	• Adjuvant T-Rez + Adebreli vs ICT in pts with residual disease • Final results not yet published
DV (HER2, MTi)	Toripalimab^a^	UC HER2+	NCT05302284 (RC48-C016)	• DV + Tor vs chemo in HER2+ UC • Like EV/P (EV-302), an approximate doubling of OS versus chemo, however, in this case with a different target antigen (HER2) (84)
DV (HER2, MTi)	Toripalimab^a^	Adv. GC HER2+	NCT05980481 (RC48-C027)	• DV + Tor + chemo (or DV + Tor + Trastuzumab) vs non-DV containing combos • Tests HER2-low-to-high subsets, with emphasis on establishing a HER2 ADC + ICI combo in the HER2-int/low setting • Updated analysis provided at ASCO (92)^&^
DV (HER2, MTi)	Tislelizumab^a^	LA/mGC/GEJC HER2-low	NCT06944496 (RC48-C039)	• DV + Tor + chemo vs Tor + chemo • HER2-low setting • Final results not yet published
DV (HER2, MTi)	Tislelizumab^a^	GC/GEJC HER2+	NCT07315750 (RC48-C040)	• DV + Tor + Trastuzumab vs Trastuzumab + P + chemo in HER2-high setting • Final results not yet published
DV (HER2, MTi)	Pembro^a^	UC HER2+	NCT05911295 (SGNDV-001)	• DV + P vs chemo/PD-L1 in HER2+ UC • Intent to replace platinum chemo regimen • Final results not yet published
DV (HER2, MTi)	Toripalimab^a^	MIBC Perioperative HER2+	NCT07608224 (HOPE-07-MIBC)	• Perioperative DV + Tori vs chemo + Tori • Similar perioperative evaluation as EV/P in MIBC, but with a different target (HER2) • Final results not yet published
DV (HER2, MTi)	Toripalimab^a^	MIBC Neoadjuvant HER2+	NCT07693959 (TJ-IRB202605029)	• Neoadjuvant DV + Tori + chemo vs DV + chemo in HER2+ setting • Final results not yet published
T-DM1 (HER2, MTi)	Atezolizumab^b^	BrC HER2+ PD-L1+	NCT04740918 (KATE3)	• T-DM1 + Atezo vs T-DM1 in HER2/PD-L1+ pts • Intent to see benefit in PD-L1-selected population relative to missed endpoint in ITT population for KATE2 • Study terminated due to low enrollment
T-DM1 (HER2, MTi)	Atezolizumab^b^	BrC Adjuvant HER2+	NCT04873362 (ASTEFANIA)	• Adjuvant T-DM1 + Atezo vs T-DM1 in HER2+ pts with neoadjuvant chemo/Trastuzumab • Final results not yet published
Iza-Bren (EGFR×HER3, TOPO1i)	Tislelizumab^a^	ESCC	NCT07554456 (PANKU-Esoph02)	• Iza-Bren + Tis ± chemo vs chemo + Tis • Intent to replace 1L chemo + Tis • Novel BiS ADC design (EGFR/HER3) • Final results not yet published
Iza-Bren (EGFR×HER3, TOPO1i)	Tislelizumab^a^	ES-SCLC	NCT07502300 (PANKU-Lung07)	• Iza-Bren + Tis ± chemo vs chemo + Tis • Intent to replace 1L chemo + Tis • Final results not yet published
BV (EGFR, MTi)	Pucotenlimab^a^	Adv. BDC EGFRmut	NCT07598318 (SRRSH2026-0432)	• BV + Puco + chemo vs Puco + chemo • EGFR mutated setting • Final results not yet published
BV (EGFR, MTi)	Pucotenlimab^a^	LA NPC High-risk	NCT07524413 (2025-FXY-502-FLK)	• BV + Puco + CRT vs Puco + CRT • Note: one of few late-stage trials assessing ADC + chemoradiotherapy (CRT) • Final results not yet published
Mo-Rez (B7-H4, TOPO1i)	Pembro^a^; Dostar^a^	EC Maintenance MMRp	NCT07684599 (BEHOLD-EC02)	• Maintenance Mo-Rez + PD-1 (ICT) vs PD-1 • Setting novelty: assesses ADC + ICI in MMRp, and as a maintenance treatment • Final results not yet published
Rina-S (FOLR1, TOPO1i)	Pembro^a^	EC MMRp	Not yet listed (NCT) (RAINFOL-08)	• Rina-S + P vs chemo + P • Tests the MMRp setting • Final results not yet published
Ris-Rez (B7-H3, TOPO1i)	Adebrelimab^b^	Adv. NSCLC No AGA	NCT07464327 (HS-20093-309)	• Ris-Rez + Adebreli vs chemo (taxane) • Final results not yet published
SV (IB6, MTi)	Pembro^a^	NSCLC PD-L1+ No AGA	NCT06758401 (Sigvie-003)	• SV + P vs P in PD-L1 TPS ≥50% pts • Final results not yet published
TV (CLDN18.2, MTi)	Tislelizumab^a^	GC/GEJC	NCT07385703 (LM302-03-201)	• TV + Tis vs chemo + Tis in PD-L1 CPS ≥ 1 and CLDN18.2+(≥75% 2+&3+) • Final results not yet published
SYS6010 (EGFR, TOPO1i)	Enlonstobart^a^	NSCLC PD-L1+	NCT07633873 (SYNSTAR 04)	• SYS6010 + Enlon vs chemo + Tis • Tests PD-L1 TPS ≥1% setting • Final results not yet published
JS107 (CLDN18.2, MTi)	Toripalimab^a^	GC/GEJC CLDN18.2+	NCT07584135 (JS107-004-III-GC)	• JS107 + Tori + chemo vs Sintilimab + chemo • Tests HER2-negative CLDN18.2+ setting • Final results not yet published

The table represents phase 2–3 ADC-ICI combinations trials in solid tumors (snapshot cutoff: September 1^st^, 2026). LA, locally advanced; mUC, metastatic urothelial carcinoma; CI, cisplatin-ineligible; CE, cisplatin-eligible; MIBC, muscle invasive bladder cancer; BC, blader cancer; Adv, advanced; AGA, actionable genetic alteration; BrC, breast cancer; TNBC, triple negative breast cancer; mBrC, metastatic breast cancer; BTC, biliary tract cancer; MMRp, mismatch repair proficient; EC, endometrial cancer; GC, gastric cancer; GEJC, gastroesophageal junction cancer; CC, cervical cancer; BDC, bile duct cancer; ESCC, esophageal squamous cell carcinoma; ES, extensive stage; mut, mutant; RT, radiotherapy; CCRT, concurrent chemoradiotherapy; TPS, Tumor Proportion Score; CPS, Combined Positive Score; SOC, standard of care; ICT, investigator choice standard therapy; PTS, patients; Pembro or P, pembrolizumab; durva, durvalumab; treme, tremelimumab (CTLA-4 ICI); rilve, rilvegostomig (PD-1/TIGIT bispecific ICI); dostar, dostarlimab; D-DXd; Dato-DXd; DV, disitamab vedotin; Iza-Bren, izalontamab brengitecan; BV, becotatug vedotin; Mo-Rez, mocertatug rezetecan; Ris-Rez, risvutatug rezetecan; SV, sigvotatug vedotin; TV, tecotabart vedotin.

^a^ PD-1 ICI; ^b^PD-L1 ICI; *or refusal of cisplatin. Green highlights represent late-stage trials with positive endpoints. Orange highlights represent discontinuations and/or failure to reach endpoints. ^&^conference-derived information. Where not referenced or specifically noted, details are derived from www.clinicaltrials.gov.

As with many clinical ADC findings, early proof of concept for ADC and immunotherapy combinations (namely, ICI) came from studies in hematological malignancies. Early phase studies evaluating brentuximab vedotin and nivolumab (PD-1 ICI) in relapsed/refractory classical Hodgkin lymphoma (cHL) revealed high overall response rates (ORR, ~>80%) with a noteworthy complete response (CR) rate of 67% and manageable safety profile (82). While these were largely single-arm studies, the combination benefit did compare favorably with historical ICI/SOC outcomes, underscoring the potential for ADC and ICI combinations. This potential was then extended to solid tumors, with enfortumab vedotin (EV, MMAE-based NECTIN-4 ADC) and pembrolizumab (anti-PD-1 ICI) demonstrating striking activity (ORR ~73%, mPFS ~12.3 months) in cisplatin-ineligible UC patients in another single-arm study (KN-869/EV-103) (83). Validation of this potential came from multi-arm studies evaluating the combination of EV and pembrolizumab in locally advanced or metastatic urothelial cancer [FDA, 2023]. In the EV-302 trial, the combination demonstrated a doubling of PFS and OS relative to SOC chemotherapy: mPFS of 12.5 months vs. 6.3 months (HR: 0.48; P<0.001), mOS of 33.8 months vs. 15.9 months; HR 0.51; P<0.001) (4, 5). Similarly, in HER2-expressing locally advanced or mUC, the combination of DV (HER2 ADC, MMAE) and toripalimab (anti-PD-1 ICI) exhibited a doubling of mPFS and OS relative to SOC chemotherapy: mPFS 13.1 months vs. 6.5 months (HR: 0.36; P<0.001), mOS of 31.5 months vs. 16.9 months (HR: 0.54; P<0.001) (84).

Not all ADC-ICI combination trials have shown clear benefit in randomized testing, particularly outside of bladder/UC. In the KATE2 trial (T-DM1 ± atezolizumab in breast cancer), the addition of the ICI did not significantly improve PFS overall (8.2 months vs 6.8 months; P = 0.33), with only a suggestive signal in PD-L1–positive tumors (8.5 months vs 4.1 months; P = 0.099) (85). This study emphasized the potential importance of using PD-L1 status to identify patients that are most likely to respond to ADC + PD-(L)1 blockade. Indeed, despite termination due to poor accrual, this information was used to facilitate the design of the KATE3 trial (**Table 2**). Influence of PD-L1 status has also been observed in other indications like NSCLC. For example, the strong survival benefit afforded by pembrolizumab in PD-L1-high (TPS ≥ 50%) NSCLC patients (KEYNOTE[KN]-024) creates a high bar for ADC-ICI combinations to compete with, most notably SG and pembrolizumab in the EVOKE-03 study – which led to trending, but non-significant improvements in survival (as reported by the trial sponsors) (86). Other studies, like TroFuse-007 (Sac-TMT + pembrolizumab) and TROPION-Lung08 (Dato-DXd + pembrolizumab) trials, have iterated on this design, requiring additional inclusion/exclusion criteria (e.g., exclusion of squamous histologies or PD-L1 TPS cutoffs) and/or alternate dosing schema (Q6W vs Q3W) for pembrolizumab. With final results for many of these late-stage comparative clinical studies maturing in the next few years, it will be critical to evaluate nuances between the myriad of factors that vary widely across indications and therapeutics. Soon, studies in (**Table 2**) may provide valuable insight into the broad applicability or context dependent (i.e., payload class, indication, target, biomarker-influenced, etc.) nature of these combinations.

In addition to well-established ADCs (e.g., EV, T-DXd, Dato-DXd, and SG), other molecules, like sacituzumab tirumotecan (Sac-TMT, MK-2870/SKB264), are providing a data-rich foundation for our understanding of ADC-immunotherapy combinations across indications. Not only are these studies providing insight into combination potential, but several are also characterizing different ADC scheduling approaches, including adjuvant, neoadjuvant, perioperative, and maintenance dosing. Trials like EV-303, EV-304, TROPION-Breast04, BEHOLD-Endometrial02 (BEHOLD-EC02) (**Table 2**) and earlier phase studies like NeoSTAR (neoadjuvant SG + pembro, NCT04230109), Neosaci-io (neoadjuvant SG + pembro, NCT05675579), and NEOVISION (neoadjuvant DV + envafolimab, NCT06734182) trials represent potentially valuable data-rich sources for future ADC and ADC-ICI trial design across indications. Additionally, several ADC-ICI combination studies have extended into settings known to be less responsive to traditional immunotherapeutic approaches, like mismatch repair proficient (MMRp) endometrial cancer, with T-DXd, Mo-Rez, Sac-TMT, and Rina-S exploring various designs for this patient populations (87). Ultimately, if these studies demonstrate benefit relative to standard of care, it will be of significant interest to elucidate the mechanistic contribution of components that enable improved immunotherapeutic responsiveness.

Given the growing confidence with ICI combinations, and enthusiasm for pairing alternative modalities, other forms of immunotherapy are beginning to filter into clinical testing; more specifically, ADCs and T cell engagers (TCE) or ‘next-generation ICI’ approaches. Combinations with dual-ICI constructs, such as the PD-1/TIGIT bispecific (rilvegostomig) have progressed into phase 3 studies (**Table 2**). However, the term ‘next-generation ICI’ refers to assets that address distinct tumor promoting pathways (e.g., immune checkpoint + angiogenic pathways), such as PD-(L)1-VEGF bispecific antibodies.

Excitement for evaluating ADC combinations with TCE and next-generation ICI comes from the previously discussed ADC-immune interplay, their promising single-agent clinical activity, and an associated expansion of competition. Despite this interest, non-clinical data that illustrates the mechanistic interplay between ADCs and TCEs/next-generation bispecifics is still limited. In recent study, Zhou et al. evaluated a TOPO1i-based ICAM-1 ADC in combination with a B7-H3-directed TCE in various *in vivo* mouse models (93). The authors found that the ADC-TCE combination not only was able to elicit significant antitumor control in various models of TNBC, but it was also able to reprogram an immune-cold TME, as characterized by increased DC maturation, infiltration/activation of CD8 T cells, and a shift of macrophage polarization. They speculated that increased ICD brought about by the combination contributed to this remodeling and supported systemic tumor control, as evidenced by inhibition of lung metastases. For PD-(L)1 bispecifics and ADC combinations, current non-clinical evidence is largely restricted to recent disclosures at major conferences (e.g., European Society for Medical Oncology, ESMO) (94).

While TCEs are more established in hematological malignancies, they are also making an impact across various solid tumor indications (e.g., SCLC and prostate cancer) (95). Tarlatamab and obrixtamig (DLL3-directed TCEs) represent two leading programs in advanced-stage clinical testing in solid tumors. Given this, it makes sense that these agents are being used as an initial testing ground for combining TCEs with ADCs in the solid tumor space. Of note, every ADC involved uses a TOPO1i payload: two that target B7H3 (risvitutatug rezetecan/ris-rez and tambotatug pelitecan/tam-peli), while one is also directed against DLL3 (**Table 3**).

**Table 3:** Snapshot of ADC and targeted immunotherapy clinical combinations (solid tumors).

ADC	TCE (all DLL3)	Indication	Phase	Trial ID	Potential learnings/Notes
SG (TROP2, TOPO1i)	Tarlatamab	ES-SCLC/NEC	1-2	NCT07328490	• Modality complementarity
Tam-Peli (HER2, TOPO1i)	Tarlatamab	ES-SCLC	1	NCT06898957 (DeLLphi-310)	• Modality complementarity • Includes a durvalumab (PD-L1) triplet group
Ris-Rez (B7H3, TOPO1i)	Tarlatamab	Multiple solids	1	NCT06551142 (EMBOLD PanTumor-101)	• Modality complementarity • Includes PK + efficacy for multiple combinations
Zoci (DLL3, TOPO1i)	Obrixtamig	Adv. SCLC/NEC	1	NCT07707895 (DAREON-36)	• Modality complementarity • Includes an atezolizumab (PD-L1) triplet group
ADC	PD-(L)1-VEGFi	Indication	Phase	Trial ID	Potential learnings/Notes
SG (TROP2, TOPO1i)	Ivonescimab^a^	TNBC	2	NCT06793332 (BALISTA)	• Focused ADC + BiS impact on brain metastases
JSKN016 (TROP2-HER3, TOPO1i)	Ivonescimab^a^	NSCLC	1	NCT06868732	• Includes an ADC + BiS + platinum triplet group
DV (HER2, MTi)	Ivonescimab^a^	CI-MIBC	1-2	NCT06957561 (DIVON)	• Perioperative setting
BV (EGFR, MTi)	Ivonescimab^a^	HNSCC	2	NCT07381075	• Neoadjuvant ADC + BiS • ADC + penpulimab (PD-1) comparator
T-Rez (HER2, TOPO1i)	Ivonescimab^a^	NSCLC	2	NCT07489703	• Tx-naïve vs 2L+ cohorts
T-DXd (HER2, TOPO1i) SG (TROP2, TOPO1i)	Ivonescimab^a^	HER2- BrC	2	NCT07394218	• ctDNA-guided study
YL202 (HER3, TOPO1i)	Pumitamig^b^	Multiple solids	1-2	NCT07070232	• Includes PK + efficacy for multiple tumor types
YL202 (HER3, TOPO1i)	Pumitamig^b^	NSCLC	1-2	NCT07111520	• Multiple PD-L1 expression and AGA cohorts
BNT3212 (EGFR-HER3, TOPO1i)	Pumitamig^b^	Multiple solids	1-2	NCT07147348	• Includes PK + efficacy
T-Pam (B7H3, TOPO1i)	Pumitamig^b^	Adv. BrC	1-2	NCT06827236	• Multiple HER2 expression cohorts
DB-1311 (B7H3, TOPO1i)	Pumitamig^b^	Multiple solids	1-2	NCT06953089	• Multiple tumor types
DB-1311 (B7H3, TOPO1i)	Pumitamig^b^	NSCLC/SCLC	1-2	NCT06892548	• Multiple subtypes/lines of lung cancer
SV (IB6, MTi) Fetra-V (PD-L1, MTi)	PF-08634404^a^	NSCLC	1-2	NCT07227298 (Symbiotic- Lung-20)	• Multiple ADCs (IB6- and PD-L1-MMAE)
EV (NECTIN-4, MTi)	PF-08634404^a^	UC	1-2	NCT07421700 (Symbiotic- GU-06)	• Includes PK + efficacy
B-Fuve (NECTIN-4, MTi)	JS207^a^	TNBC	2	NCT07045311	• BiS + chemo comparator
JS212 (EGFR-HER3, TOPO1i)	JS207^a^	NSCLC/SCLC	2	NCT07309276	• Includes ADC + PD-1-IL-2 BiS and ADC + PD-1 groups
JS212 (EGFR-HER3, TOPO1i)	JS207^a^	Multiple solids	2	NCT07480733	• Includes ADC + PD-1-IL-2 BiS and ADC + PD-1 groups
DV (HER2, MTi) RC88 (MSLN, MTi)	RC148^a^	Multiple solids	1-2	NCT06016062	• Multiple chemo and ADC + BiS combos
RC118 (CLDN18.2 ADC, MTi)	RC148^a^	Multiple solids	1-2	NCT06038396	• Includes a PD-1 triplet group
DV (HER2, MTi)	RC148^a^	HER2-low BrC	1-2	NCT06642545	• PD-1 comparator group
EV (NECTIN-4, MTi) T-DXd (HER2, TOPO1i) Sac-TMT (TROP2, TOPO1i)	CVL006^b^	Multiple solids	1-2	NCT07157956	• Multiple chemo and ADC + BiS combos
B-Fuve (NECTIN-4, MTi)	CVL006^b^	Multiple solids	1-2	NCT07615660	• Includes PK + efficacy

The table represents a snapshot of early phase ADC and targeted immunotherapy combinations trials in solid tumors (snapshot cutoff: September 1^st^ 2026). Here, targeted immunotherapy focuses on TCEs and representative PD-(L)1-VEGFi bispecifics (BiS). ^a^PD-1-VEGFi BiS; ^b^PD-L1-VEGFi BiS.

PK, pharmacokinetics; NEC, neuroendocrine cancer; CI, cisplatin-ineligible; Tx, therapy; Tam-Peli, tambotatug pelitecan, Zoci, zocilurtatug pelitecan; DV, disitamab vedotin; BV, becotatug vedotin; T-Pam, trastuzumab pamirtecan; fetra-v, fetrastobart vedotin; SV, sigvotatug vedotin; B-Fuve, bulumtatug fuvedotin. Where not referenced or specifically noted, details are derived from www.clinicaltrials.gov.

A growing number of clinical studies underscore the potential for PD-(L)1-VEGF bispecific antibodies (e.g., HARMONI-6, ROSETTA Lung-02, etc.) (96). As such, efforts for PD-(L)1-VEGF bispecifics are more expansive, with multiple assets, indications, and ADCs under early phase clinical evaluation (**Table 3**). Like ADC-ICI combinations, questions remain regarding impact of dose scheduling (*viz*, is dose sequencing of ADCs and TCEs necessary for optimal benefit – and, if so, in what orientation)?. The design for many of these combination studies appears to be more traditional, leveraging concomitant dosing, therefore future studies may be needed to address these questions.

Lastly, with any combination regimen, regardless of the level of predicted mechanistic overlap, attention must be paid to emergent toxicities. Recent meta-analyses have been conducted to explore the clinical benefits and toxicities associated with ADC + immunotherapy combinations (28, 97, 98). At a high level, ADC and ICI combinations exhibit similar rates of the most common toxicities relative to single agents. With that said, even though they are often manageable, numerical increases in these AEs are often observed (85, 99). Also, given the amount of variegation in ADC design, payload characteristics (e.g., bystander activity, potency, etc.), targets/mechanisms, and underlying disease heterogeneity, these analyses may not provide a universal truth for emergent toxicities across ADC and immunotherapy combinations. As discussed previously, attention should be paid to context-specific toxicities and ADC design characteristics that may be traced to immune populations, such as ILD/pneumonitis or even rash.

## Concluding perspectives

The evidence reviewed here supports the view that ADCs can possess immunomodulatory properties that may contribute to therapeutic activity and toxicity in a context-dependent manner. As noted with T-DXd, the immunological dimension appears mechanistically linked to an ADC’s therapeutic activity and safety profile. Despite a greater appreciation for the immunologic component of the TME as a hallmark of cancer, the field’s dominant framework for understanding ADC mechanism (e.g., antigen binding, internalization, enzymatic processing, and cytotoxicity) does not or is just starting to account for immunomodulation as a potential byproduct of therapy (6–8, 100). Antibody isotype, Fc engineering, linker chemistry, and payload class are typically optimized against potency, stability, and target engagement, with immunological consequences assessed after the fact, if at all. An analysis of patritumab deruxtecan (P-DXd) in the ICARUS-Breast01 phase 2 study demonstrated that baseline vs. on-treatment TME characterization is complex and influenced by the site and timing of biopsy as well as intratumoral ADC distribution (101). Future studies of this type may employ interferon gene signatures in circulating tumor cells as a surrogate for the study of the ADC-tumor cell interaction and prediction of treatment response.

Optimizing the immunologic risk-to-benefit ratio of ADCs, in the midst of a rapidly evolving landscape, will require a paradigm shift in how these molecules are designed and evaluated. First, Fc engineering decisions should be treated as immunological design choices rather than purely PK or bioconjugation considerations. The limited clinical experience with Fc-enhanced ADCs, concentrated in hematologic malignancies, leaves open fundamental questions about whether afucosylation or FcγR-selective mutations would benefit or detract from therapeutic potential in solid tumors. Non-clinical safety models that systematically vary Fc format against matched linker-payloads, particularly for TOPO1i-based ADCs, would help separate Fc-mediated from payload-driven toxicity, a distinction that remains obscured in current clinical comparisons.

Second, WLP design should include ICD induction as a prospective design parameter. Most ICD characterization to date has been payload-centric by broadly documenting DAMP release or APC activation after the selection of a WLP construct. Next-generation formats including dual-payload ADCs, DACs, and novel payload classes such as NMT inhibitors, are being developed largely on cytotoxic potency, with ICD potential poorly characterized if at all. Given the apparent contribution of ICD to durable antitumor responses and combination benefit, incorporating ICD endpoints earlier in payload testing and selection, rather than as a *post hoc* rationalization for clinical activity, could meaningfully change which molecules advance.

Third, the field needs better tools for anticipating on-target and bystander immune toxicity before clinical testing. Predictive nonclinical models of ILD risk remain a gap limiting a systematic and empirical approach to warhead-linker-payload design to mitigate this known risk for TOPO1i-based ADCs. In addition, the recurring pattern of hematopoietic and TME-resident immune cell depletion, from CD56-directed NK cell loss to T cell bystander killing in “hot” tumors, argues for incorporating immune cell vulnerability, not just tumor antigen expression, into target and linker selection. This is particularly relevant for indication expansion of existing molecules and for targets with meaningful stromal or immune-lineage expression (e.g., B7H3).

Finally, the maturing landscape of ADC and immunotherapy combinations, spanning ICI, TCE, and next-generation bi/trispecifics, offers a real opportunity to test the immunological hypotheses raised throughout this review, but only if trials are designed to interrogate mechanism rather than simply measure clinical outcomes. Dose sequencing, scheduling (neoadjuvant, adjuvant, maintenance), and biomarker-driven patient selection remain largely unresolved, and mixed results outside urothelial cancer (e.g., KATE2) suggest that combination benefit is context-dependent rather than universal. Meta-analyses across the growing set of TOPO1i-based ADCs sharing common targets (e.g., HER2, B7H3) represent a near-term, tractable opportunity to disentangle the relative contributions of antibody, linker, and payload to both efficacy and immune toxicities, insight that will be difficult to obtain from any single asset in isolation.

Taken together, ADCs sit at the intersection of targeted therapy and cancer immunology. Realizing their full potential, and managing their risks, will depend on treating immune engagement as a designable property of the molecule rather than a secondary downstream consequence of treatment to be explained following clinical observation – and while this concept is intriguing, the current pool of evidence has notable gaps that need to be filled. Much of the mechanistic data remains either speculative or preclinical, generated in models that may not directly translate to effects in cancer patients. Consequently, while ADC and immunotherapy combination trials grow, clinical studies designed to specifically evaluate ADC pharmacodynamic effects and immune biomarkers are needed to evolve our understanding around ADC and immune interplay.
